# Supplementary material for: Mechanisms of Calorie Restriction: A Review of Genes Required for the Life-Extending and Tumor-Inhibiting Effects of Calorie Restriction
Source: Nutrients. 2019 Dec 16;11(12):3068. doi: 10.3390/nu11123068 (PMC6950657; doi:10.3390/nu11123068)
Supplement: Supplementary file 1 [file nutrients-11-03068-s001.pdf]

Supplemental Table 1. Pathways signified by differentially regulated genes between WT-CR and *Foxo3*$^{+/-}$ CR livers in the fasting phase.

Pathways	pValue	Genes	Predicted Direction
Role of NFAT in Regulation of the Immune Response	0.001309	PLCB1, PLCG1, AKT3, CD80, NFAT5, CAMK4, JUN, GNAO1, HLA-DOB, GNB3, MS4A2	Varied
Chemokine Signaling	0.004402	Ppp1r12b, PTK2, JUN, CAMK4, PLCB1, PLCG1	Up
Cellular Effects of Sildenafil (Viagra)	0.007183	Ppp1r12b, KCNQ2, CAMK4, MYH3, PDE5A, PLCB1, PLCG1, CACNG8	Varied
iCOS-iCOSL Signaling in T Helper Cells	0.009221	CAMK4, NFAT5, CD80, HLA-DOB, AKT3, PLCG1, ICOSLG/LOC102723996	Up
Role of Macrophages, Fibroblasts and Endothelial Cells in Rheumatoid Arthritis	0.013715	CAMK4, IL1RAPL2, FZD3, PLCG1, ROR2, NFAT5, JUN, WNT10A, GNAO1, AKT3, PLCB1, LEF1, LRP1	Up
CD28 Signaling in T Helper Cells	0.014532	JUN, CAMK4, NFAT5, CD80, HLA-DOB, AKT3, PLCG1	Up
Gαq Signaling	0.015054	HTR2C, GNB3, CAMK4, Agtr1b, PLCB1, AKT3, PLCG1, FNBP1	Up
Agrin Interactions at Neuromuscular Junction	0.016987	PTK2, CHRNA1, JUN, UTRN, NRG4	Varied
CXCR4 Signaling	0.01806	PTK2, GNB3, JUN, EGR1, GNAO1, PLCB1, AKT3, FNBP1	Varied
D-myo-inositol (1,4,5)-Trisphosphate Biosynthesis	0.020182	PLCB1, PLCG1, PIP5K1B	Up
PI3K Signaling in B Lymphocytes	0.021731	JUN, CAMK4, NFAT5, FOXO3, PLCB1, AKT3, PLCG1	Up
Thrombin Signaling	0.023947	Ppp1r12b, PTK2, GNB3, CAMK4, GNAO1, PLCB1, AKT3, PLCG1, FNBP1	Up
Regulation of IL-2 Expression in Activated and Anergic T Lymphocytes	0.028639	JUN, CAMK4, NFAT5, CD80, PLCG1	Up
Wnt/β-catenin Signaling	0.031481	JUN, WNT10A, FZD3, GNAO1, SOX12, AKT3, LEF1, LRP1	Varied
G Protein Signaling Mediated by Tubby	0.034238	GNB3, PLCB1, PLCG1	Varied
DNA Double-Strand Break Repair by Homologous Recombination	0.035996	GEN1, BRCA1	Varied
B Cell Development	0.036964	CD80, IGHM, HLA-DOB	Varied
Glioblastoma Multiforme Signaling	0.040292	WNT10A, FZD3, PLCB1, AKT3, PLCG1, LEF1, FNBP1	Up
nNOS Signaling in Skeletal Muscle Cells	0.040949	CHRNA1, CAMK4	Varied
Protein Kinase A Signaling	0.041655	PTPRE, CAMK4, CNGA4, MYLK3, PLCG1, PPP1R3A, PTK2, NFAT5, GNB3, PDE5A, PLCB1, LEF1, ANAPC11, PTPRT	Varied
P2Y Purigenic Receptor Signaling Pathway	0.04533	P2RY4, GNB3, JUN, PLCB1, AKT3, PLCG1	Varied
Sperm Motility	0.046884	PTK2, CNGA4, CAMK4, PLCB1, PLCG1, PLA2G4F	Up

Supplemental Table 2. Pathways signified by differentially regulated genes between WT-CR and *Foxo1*$^{+/-}$ CR livers in the fasting phase.

Pathways	p Value	Genes	Predicted direction
Nur77 Signaling in T Lymphocytes	0.0103	CAMK4, HLA-DQA1, HLA-DQB1, HLA-DRB5	Down
Acyl-CoA Hydrolysis	0.0133	ACOT2, ACOT1	Down
B Cell Development	0.0140	HLA-DQA1, HLA-DQB1, HLA-DRB5	Down
Melatonin Degradation III	0.0149	MPO	Up
Calcium-induced T Lymphocyte Apoptosis	0.0153	CAMK4, HLA-DQA1, HLA-DQB1, HLA-DRB5	Down
Antigen Presentation Pathway	0.0176	HLA-DQA1, CD74, HLA-DRB5	Down
Role of Oct4 in Mammalian Embryonic Stem Cell Pluripotency	0.0311	FOXA2, CDX2, SALL4	Up
Graft-versus-Host Disease Signaling	0.0347	HLA-DQA1, HLA-DQB1, HLA-DRB5	Down
Autoimmune Thyroid Disease Signaling	0.0366	HLA-DQA1, HLA-DQB1, HLA-DRB5	Down
Allograft Rejection Signaling	0.0398	H2-T3, HLA-DQA1, HLA-DQB1, HLA-DRB5	Down
FXR/RXR Activation	0.0418	FOXO1, SREBF1, FOXA2, VLDLR, FGA	Varied
OX40 Signaling Pathway	0.0442	H2-T3, HLA-DQA1, HLA-DQB1, HLA-DRB5	Down
